# Adenoid cystic carcinoma of the peripheral lung: a case report

**DOI:** 10.1186/1477-7819-8-74

**Published:** 2010-08-26

**Authors:** Masahiro Kitada, Keisuke Ozawa, Kazuhiro Sato, Satoshi Hayashi, Yoshihiko Tokusashi, Naoyuki Miyokawa, Tadahiro Sasajima

**Affiliations:** 1 Department of Surgery, Asahikawa Medical University, Midorigaoka-Higashi 2-1-1-1 Asahikawa Hokkaido 078-8510, Japan; 2 Department of Clinical Pathology, Asahikawa Medical University, Midorigaoka-Higashi 2-1-1-1 Asahikawa Hokkaido 078-8510, Japan

## Abstract

Adenoid cystic carcinoma of the peripheral lung is a rare entity. We recently encountered a patient with adenoid cystic carcinoma. A 75-year-old woman showed a nodular lesion with 10 mm in diameter in the right upper lung field on chest radiography. The diagnosis was unclear, but lung cancer could not be ruled out. Thoracoscopic biopsy was performed, and intraoperative pathological diagnosis revealed the carcinoma of the lung. We enforced upper lobectomy and mediastinal lymph node dissection to the patient. Histopathological examination revealed adenoid cystic carcinoma with a characteristic cribriform structure. Immunohistochemical examination revealed that the tumor cells were positive for thyroid transcription factor 1 (TTF-1), this tumor was diagnosed primary ACC of the lung.

## Background

Adenoid cystic carcinoma (ACC) of the lung is a relatively rare lung cancer arising from the bronchial glands and accounting for about 0.04-0.2% of all lung cancers [1,2]. ACC has a slow growing and prolonged clinical course, and thus is considered a low-grade malignancy. The site of origin is the tracheobronchial glands, so ACC is more common in the central bronchi than in the segmental bronchi. Reports of ACC originating in the peripheral lung are rare. In addition, in cases of occurrence in the periphery, lung metastases from a salivary gland tumor must be ruled out. It reports a case of ACC which we experienced.

## Case

A 75-year-old woman showed a nodular lesion in the right upper lung field on the chest radiography with 6 mm diameter. After 1 year of observation, follow-up chest radiography showed an increase in size to 10 mm. In the thorough examination of the bronchoscope, the definitive diagnosis wasn't on, so the patient was introduced to our department for diagnosis and treatment.

Past medical history included hypertension and diabetes mellitus treated with oral medications. In addition, a salivary gland tumor had been resected 19 years ago, but details were unknown. Family history was unremarkable, and she had never been a smoker. Her height was 149 cm, the weight was 44 kg, hers blood pressure was 135/91 mmHg and hers heart rate was 65 beats/min (normal sinus rate). She had no abnormal findings during the physical examination. The biochemical examination of blood did not revealed abnormalities, and levels of tumor markers carcinoembryonic antigen (CEA) was 2.9 ng/ml, squamous cell carcinoma antigen (SCC) was 0.6 ng/ml, Cyfra21-1/cytokeratin 19 fragment (CYFRA) 0.96 ng/ml, and neuron-specific enolase (NSE) was 3.2 ng/ml.

**Figure 1:**
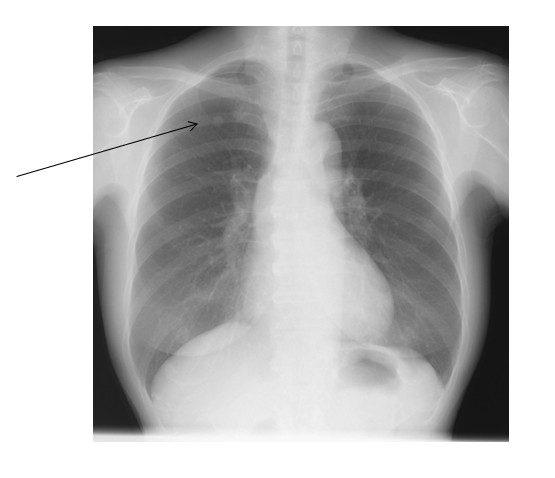
**Chest radiography showing a nodular shadow in the upper lung field**.

**Figure 2:**
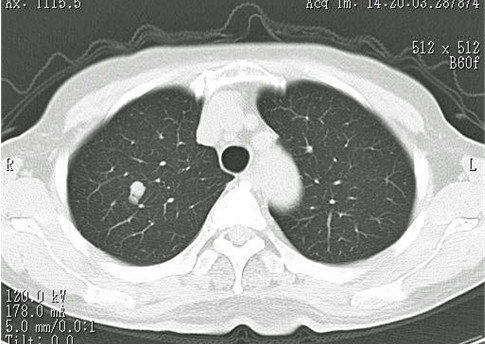
**Chest CT showing the tumor, 10 mm in diameter, in S1**.

Pulmonary function tests showed that vital capacity (VC) was 2520 ml, percentage of predicted VC was 117.8%, forced expiratory volume in 1 s (FEV1) was 1780 ml, and percentage of predicted FEV1 was 74.2%. Chest radiography revealed a well circumscribed, rounded tumor nodule in the right upper lung field (Fig. 1). Chest computed tomography (CT) demonstrated a partially serrated border solid nodule 10 mm in diameter nodular in the right S1 region (Fig. 2). On bronchoscopy, no abnormalities were seen in the visualized area, and cytology revealed no malignant cells. Malignancy could not be excluded based on these findings and including increasing size, so surgery was planned.

**Figure 3:**
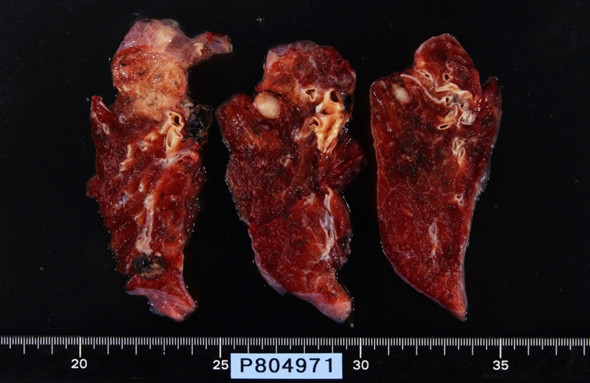
**Macroscopic findings showing a tumor with clear boundaries, a uniform 10 mm in diameter**.

**Figure 4:**
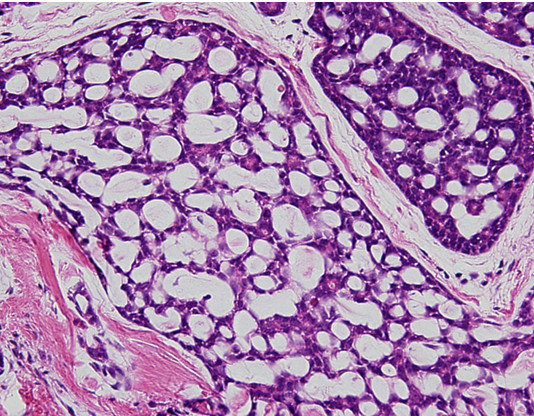
**Histological findings showing typical cribriform pattern and constituent cells including of bronchial epithelial cells, myoepithelial cells, and basal cells (HE ×400)**.

**Figure 5:**
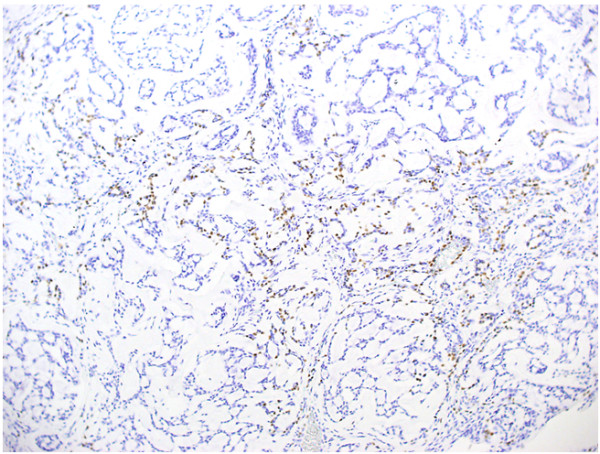
**Immunohistochemical examination revealed that tumor cells were positive for TTF-1 (×100)**.

**Figure 6:**
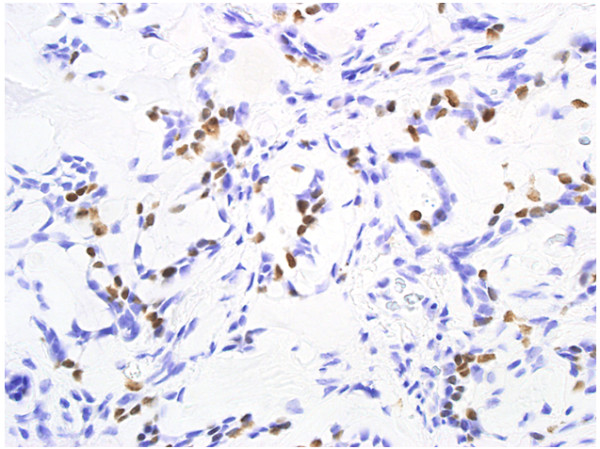
**Immunohistochemical examination revealed that tumor cells were positive for TTF-1(×400)**.

A mini thoracotomy was made in the 5th intercostal space, and the mass was biopsied under video-assisted thoracoscopic surgery. Intraoperative pathological diagnosis showed mucinous carcinoma. Standard upper lobectomy and mediastinal lymph node dissection (ND2a) was performed. The macroscopic specimen (Fig. 3) showed a 15-mm (maximum diameter) mass with a nearly uniform internal structure. Histopathological examination (Fig. 4) with hematoxylin and eosin (HE) staining showed ACC with a cribriform structure and mucinous component. On immunohistochemical analysis, these tumor cells were positive for TTF-1(Fig. 5 (×100) and Fig. 6 (×400). This tumor was diagnosed primary ACC of the lung and p-T1N0M0 stage 1A. Because of surgical margin was free, it didn't do radiotherapy and chemotherapy. Postoperative course was satisfactory, and the patient was discharged on hospital day 12. At present, as of 2 years after surgery, no signs of recurrence have been identified.

## Discussion

ACC of the lung is a malignant tumor arising in the tracheobronchial glands distributed in the airway submucosa, with a morphology similar to ACC arising in the salivary glands. In general, as with typical carcinoid and mucoepidermoid tumors, this lung tumor shows low grade malignancy. ACC represents 0.04-0.2% of all lung cancers [1,2], and >90% arise in the central rather than segmental bronchi, thus accounting for the small number of reported cases in the peripheral lung [3]. ACC in the peripheral lung must therefore be distinguished from metastatic lung tumor.

Our patient had undergone resection of a salivary gland tumor 19 years previously, although the details are unknown. Metastatic lung tumor therefore had to be ruled out. For this purpose, immunostaining for TTF-1 is useful to diagnose a lung primary lesion. TTF-1 is highly sensitive and specific for diagnosis of primary lung cancer. TTF-1 is a transcription factor specifically expressed in the thyroid and lung, and is expressed in 60-70% of pulmonary adenocarcinomas. TTF-1 is expressed in follicular epithelial cells of the thyroid, and in type II alveolar epithelial cells and Clara cells of the lung. TTF-1 is expressed in pulmonary adenocarcinomas, but not in metastatic lung tumors (except thyroid tumor metastases), so this marker is useful to distinguish between primary and metastatic lesions [4,5]. In our patient, TTF-1 was positive and no thyroid lesions were present, thus establishing a final diagnosis of primary lung cancer.

The first-line treatment for ACC is surgical resection. As ACC often arises in the central airways, tracheobronchoplasty is also often indicated [6,7]. In addition, although the tumor may appear localized, the resection margins may show positive results for tumor cells due to widespread invasion along the airway. However, radiation sensitivity is relatively high, so radiotherapy may be useful [7,8]. Similarly, bronchial submucosal progression may be present even in peripheral lesions, so intraoperative pathological diagnosis has been recommended. In this ACC, the tumor showed a small diameter and seemed completely resectable, so intraoperative pathological diagnosis was not performed for the bronchial margins. Bronchial margins of the resected specimen proved negative for malignant cells, but evaluation should probably be performed for similar cases in future. In addition, because ACC is a low grade malignancy, long term survival can be expected. However, recurrences after ≥10 years are not uncommon, so careful follow-up observation is mandatory.

## Consent statement

Informed consent was obtained from the patient for publication of this case report and accompanying images. A copy of the written consent is available for review by the Editor-in-Chief of this journal.

## Competing interests

The authors declare that they have no competing interests.

## Authors' contributions

MK have operated this case and analyzed all data. KO, KS, SH did the assistant of the operation. YT and NM diagnosed the pathology of this case. TS was the professor of the surgical science and had a guide.
